# Sustainable Treatment of Oral Traumatic Ulcers with Licorice Containing Hydrogels: Integrating Computational Modeling, Quality by Design, Green Synthesis, and Molecular Biological Evaluation

**DOI:** 10.3390/pharmaceutics15122734

**Published:** 2023-12-06

**Authors:** Sarah G. Moussa, Nada M. El Hoffy, Yara Y. Mouselhy, Ramy Mubarak, Reem T. Attia, Noha Khalil, Sherif A. Amer

**Affiliations:** 1 Department of Oral Medicine, Periodontology, and Diagnosis, Faculty of Oral and Dental Medicine, Future University in Egypt, Cairo 11835, Egypt; sara.gamal@fue.edu.eg (S.G.M.); ramy.mobarak@fue.edu.eg (R.M.); sherif.abdelrahman@fue.edu.eg (S.A.A.); 2 Department of Pharmaceutics and Pharmaceutical Technology, Faculty of Pharmacy, Future University in Egypt, Cairo 11835, Egypt; 3 Department of Oral Pathology, Faculty of Oral and Dental Medicine, Future University in Egypt, Cairo 11835, Egypt; yara.youssef@fue.edu.eg; 4 Department of Pharmacology and Toxicology and Biochemistry, Faculty of Pharmacy, Future University in Egypt, Cairo 11835, Egypt; reem.tarek@fue.edu.eg; 5 Department of Pharmacognosy and Medicinal Plants, Faculty of Pharmacy, Future University in Egypt, Cairo 11835, Egypt; noha.hassan@fue.edu.eg

**Keywords:** green synthesis, sustainability, licorice, oral ulcer, hydroxyethyl cellulose, hydrogels

## Abstract

The urge to implement innovative approaches that align with eco-friendly practices and hold promise for enhancing oral health while promoting environmental sustainability has been increasing. This current work aims to develop a sustainable treatment for oral traumatic ulcers using licorice-based hydrogels (LHGs) containing hydroxyethyl cellulose (HEC) as the green gelling agent. Licorice root aqueous extract was phytochemically profiled using UPLC-ESI-MS/MS. Forty-three compounds were detected, with Glycyrrhizic acid being the major component of the extract (34.85 ± 2.77%). By implementing a Quality by Design (QbD) approach, the study investigates the effects of different licorice extract and HEC concentrations on key variables such as pH and viscosity of the prepared formulations, ulcer and wound healing scores, and tissue growth factors via a Full Factorial Experimental Design. The LHGs exhibited desirable consistency, spreadability, and clarity. Statistical analysis, employing an ANOVA test, revealed the high significance of the constructed models with the licorice concentration being the key independent factor affecting all dependent outputs. The pH as well as the viscosity of the prepared LHGs were positively influenced by licorice extract concentration, with higher concentrations leading to increased alkalinity and viscosity. Rheological behavior analysis revealed a pseudoplastic flow with demonstrated thixotropy which is advantageous for application and prolongation of residence time. The wound healing process was assessed through ulcer size, traumatic ulcer healing score (UHS), collagen-1 expression (COL-1), growth factors (EGF, VEGF), pro-inflammatory markers (TNF-α), wound healing score (WHS). LHGs prepared using higher levels of both factors, 30% dried licorice root extract and 4% HEC, demonstrated enhanced wound healing, elevated growth factor expression of 66.67% and 23.24%, respectively, and 88% reduced inflammation compared to the control group, indicating their potential in expediting oral ulcer recovery. Overall, these findings highlight the promising role of green licorice-based hydrogels in promoting sustainable oral mucosal healing.

## 1. Introduction

Traumatic ulcers in the oral cavity typically result from mechanical, chemical, thermal, or electrical injuries. They are often found on nonkeratinized surfaces like the buccal/labial mucosa, the tongue’s lateral borders, lips, and the soft or hard palate [1]. These ulcers involve the loss of the epithelial layer extending beyond the basement membrane and can be painful, disrupt chewing, impact nutrient intake, and reduce the overall quality of life [2,3]. The prevalence of traumatic ulcers is relatively common, affecting approximately 3–24% of the population [4]. Furthermore, the dynamic oral cavity environment, containing various microorganisms, exacerbates oral ulcer healing, requiring traumatic treatment to reduce pain and inflammation and accelerate healing [5,6].

Green treatments for oral diseases offer numerous advantages that align with global sustainability goals. Firstly, these eco-friendly approaches utilize natural and biodegradable materials like natural plant extracts and natural polymers, reducing the environmental impact caused by traditional chemical-laden treatments [7,8]. By incorporating organic ingredients and promoting minimal waste generation, these practices contribute to the goal of conserving resources and protecting biodiversity [9]. Moreover, embracing green dental practices fosters innovation in research and technology, encouraging the development of sustainable materials and processes that can be applied beyond oral care, and contributing to the broader global efforts towards achieving the UN Sustainable Development Goal number nine on industry, innovation, and infrastructure. Overall, green treatments for oral diseases offer a promising path towards a healthier, environmentally conscious, and sustainable future.

Since the prehistoric era, licorice has been used as a successful phytochemical in the treatment of various diseases affecting the respiratory, gastrointestinal, cardiovascular, and urogenital systems. In addition, its strong anti-inflammatory effect provides promising outcomes in the treatment of atopic dermatitis [10]. *Glycyrrhiza glabra* L. (licorice) extract has shown many compounds with strong proven anti-inflammatory and antioxidant effects, namely: Glycyrrhizin, glabridin, glabrene, liquiritigenin, isoliquiritigenin, and polysaccharides. In addition to the reduction in cellular oxidative stress and its malondialdehyde marker, licorice extracts significantly boosted neutrophils’ chemotactic potentials as well as the total and differential leukocyte counts. It promoted wound healing by enhancing complete re-epithelialization, collagen synthesis, and angiogenesis [11].

The effect of licorice and its metabolites in inhibiting and treating multiple oral diseases such as periodontal diseases, dental caries, aphthous ulcers, candidiasis, and debilitating diseases like oral cancer has been in focus recently [12]. Its topical application to diseased periodontal sites can be useful due to its action on the periodontopathogens and the host inflammatory response. In addition, buccal mucoadhesive licorice films were reported to have respectable anti-inflammatory activity and an anti-microbial effect [12]. Moreover, evidence exists for the successful use of licorice extract in different forms (paste, patch, and mouthwash) in the management of recurrent aphthous stomatitis, enhancing ulcer healing without demonstrating any adverse effects [13,14]. Additionally, licorice mouthwash was successfully used as an herbal replacement for chlorhexidine mouthwash [15] at different mouth wash concentrations (1% or 5%) [16]. To the best of our knowledge, the clinical, histological, and biochemical effects of licorice extract on the healing of oral traumatic ulcers have not been investigated in the literature. Moreover, information about its effectiveness in various concentrations in hydrogel form is scarce.

Hydrogels derived from natural sources, such as hydroxyethyl cellulose (HEC) hydrogels, present significant advantages in the field of oral disease treatments while also aligning with global sustainability goals [17]. Hydroxyethyl cellulose is a biocompatible and non-toxic compound, making it safe for oral use and reducing the potential risk of adverse effects on patients. These hydrogels have excellent water retention properties, providing a moist environment that aids in tissue healing and regeneration, which is particularly beneficial for oral wounds and ulcers [18]. By utilizing renewable and biodegradable resources, HEC hydrogels contribute to the reduction of plastic waste and the overall environmental impact associated with conventional synthetic materials. Additionally, their natural origin aligns with sustainability objectives by promoting the preservation of biodiversity and ecosystem health.

Moreover, HEC exhibits remarkable antimicrobial and anti-inflammatory effects, making it a promising candidate for various biomedical applications, including oral disease treatments [19]. When formulated into hydrogels, HEC can release active agents slowly, creating a controlled release system that inhibits the growth and proliferation of microorganisms, such as bacteria and fungi. This property is particularly valuable in oral care, where the mouth harbors a diverse range of microorganisms that can lead to dental caries, periodontal diseases, and other oral infections [20]. HEC hydrogels can act as a protective barrier, preventing the attachment and colonization of harmful bacteria on teeth surfaces or oral tissues, thus maintaining oral health and preventing the progression of oral diseases.

Although inflammation is a crucial aspect of the body’s immune response, chronic inflammation can lead to tissue damage and exacerbate various oral diseases [21]. HEC hydrogels can help control and modulate inflammatory responses by reducing the release of pro-inflammatory cytokines and other mediators [22]. When applied to inflamed oral tissues, HEC hydrogels can provide a soothing and calming effect, alleviating pain and discomfort associated with oral conditions like gingivitis and mucositis. By mitigating inflammation, HEC hydrogels promote the healing process and support tissue repair, aiding in the recovery from oral injuries and surgeries. All of which makes it a pioneering candidate for the green treatment of oral inflammatory conditions such as oral ulcers.

Wound healing is a dynamic process involving the orchestrated interplay of collagen production, the controlled regulation of pro-inflammatory cytokines like TNF-alpha, and the expression of growth factors such as EGF [21,23]. The precise balance of these mechanisms is crucial for efficient wound healing and the restoration of tissue structure and function. Thus, investigating the ability of the treating moiety to augment the dominance of the expression of these markers presents a crucial breakthrough.

Quality by Design (QbD) is a systematic approach used in research and development across industries like pharmaceuticals, biotechnology, and manufacturing. It emphasizes a science-based understanding of the product and process, focusing on optimizing processes to reduce waste, minimize resource consumption, and increase efficiency, which enables the design of sustainable products from the beginning. QbD also encourages a risk-based approach, identifying and addressing potential challenges early in the research process to avoid costly setbacks. This aligns with sustainability principles, minimizing the negative impact of research activities on the environment and ensuring resource use wisely. Moreover, QbD promotes continuous improvement and optimization throughout the research lifecycle, using data-driven decision-making processes to identify opportunities for improvement and make iterative adjustments. This pursuit of excellence aligns with sustainability, encouraging ongoing efforts to reduce waste, conserve resources, and enhance research efficiency [24,25].

The objective of the present study is the sustainable treatment of traumatic oral ulcers through the green synthesis of licorice-based hydrogels with different concentrations of the extract, utilizing HEC as the green gelling agent in favor of both its antimicrobial as well as its anti-inflammatory effects. In addition, the deciphering of the intracellular mechanistic pathway underlying the wound healing potential of licorice through exploring its action on inflammation, cell proliferation, re-epithelialization, collagen synthesis, and angiogenesis was also studied. The QbD approach was adopted through the implementation of a Full Factorial Experimental Design employing Design Expert© 11 to study the effect of the independent variables on the dependent ones, thus preserving environmental resources and ensuring sustainability.

## 2. Materials and Methods

### 2.1. Materials

Hydroxy ethyl cellulose was a kind gift from Lit and Glow Co., obtained from CISME, Italy. Dried roots of *Glycyrrhiza glabra* L. were a gift from Lit and Glow Co. obtained from Harraz Farm and Garden, Egypt. Rat-EGF (Epidermal Growth Factor) ELISA Kit was purchased from Elabscience Biotechnology Co., Ltd., Houston, TX, USA; Catalog #: E-EL-R0369, VEGF ELISA Kit was obtained from Cloud-Clone Corp, Houston, TX, USA; Catalog # SEB851Ra, COL-1 ELISA Kit was bought from MyBioSource, Inc., San Diego, CA, USA; Catalog #: Cat No. MBS262647 and TNF-α ELISA Kit were also purchased from MyBioSource, Inc., San Diego, CA, USA; Catalog #: MBS2507393.

### 2.2. Methods

#### 2.2.1. Preparation of Licorice Extract

The roots of *Glycyrrhiza glabra* L. were collected from the medicinal farm of Harraz Farm and Garden, authenticated by MS Therese Labib, who is a botanical specialist and consultant at Orman Botanical Garden, Giza, Egypt, and dried by the same cultivator. Five hundred g of *Glycyrrhiza glabra* L. roots were washed under running tap water for 15 min and then dried in shade for 4 days. The dried rhizomes were then pulverized and extracted with distilled water in a ratio of 1:10% *w*/*v*. This was followed by centrifugation at 4000 rpm for 20 min, then filtration and evaporation of the solvent under reduced pressure using a rotary evaporator. The obtained dried extract (59 g) was kept in sealed vials at 4 °C until analysis. An exact amount of dried licorice roots (*Glycyrrhiza glabra* L.) was added to the precisely measured distilled water as shown in Table 1 to obtain the desired concentration [26,27,28].

#### 2.2.2. Identification of Metabolites in Licorice Extract Using UPLC-ESI-MS/MS

The dried aqueous extract of Licorice roots was analyzed using ultra-performance liquid chromatography-electrospray tandem mass spectrometry (UPLC-ESI-MS/MS). The analysis was carried out in negative ion acquisition mode on a Triple Quadrupole Mass Spectrometer (XEVO TQD, Waters Corporation, Milford, MA, USA). Sample solutions (100 μg/mL in HPLC-grade methanol) were filtered through a 0.2 μm PTFE membrane disc filter. Degassing was carried out by sonication. The injection volume was 10 μL. A reverse-phase C18 column was used (ACQUITY UPLC-BEH C18, 1.7 µm particle size, 2.1 × 50 mm column). The mobile phase consisted of eluent A (0.1% formic acid in methanol) and eluent B (water acidified with 0.1% formic acid). The elution was a gradient with a flow rate of 0.2 mL/min as follows: 10% A (0–0.3 min), 10–90% A (0.3–18 min), 90% A (18–22 min), and 10% A (22–25 min). Negative ion ionization mode was used (mass spectra detected between *m*/*z* 100–900) with source temperature of 150 °C, capillary voltage of 3 kV, cone voltage of 30 eV, de-cone gas flow, 50 mL/h, solvation temperature of 450 °C, and de-solvation gas flow of 900 L/h. MasslynxTM 4.1 software (Waters Inc., Millford, MA, USA) was used. Compounds were tentatively identified based on their molecular weight, fragmentation pattern of the mass spectrum, and comparison with previously published data. The relative peak area of each compound was calculated to indicate the relative percentage of the extract components.

#### 2.2.3. Preparation of Licorice-Based Hydrogels (LHGs)

An accurate amount of the natural biocompatible polymer hydroxy ethyl cellulose was weighed and dissolved directly into exactly measured amounts of the aqueous licorice extracts and/or distilled water, as shown in Table 1, and kept under stirring for 1 min at 25 °C until complete dissolution of the gelling agent and obtaining the hydrogels. The prepared LHGs were subjected to physical examination concerning their clarity, texture, stiffness, and spreadability.

#### 2.2.4. Design of Experiment (DOE) and Construction of the 2^1^.3^1^ Full Factorial Experimental Design

DOE was utilized for the generation and evaluation of the obtained models for the formulation of LHGs using Design Expert^®^ 11 software (Stat-Ease, Minneapolis, MN, USA). A 2^1^.3^1^ full factorial experimental design was computed to investigate the joint effect of independent formulation variables on the characteristics of the prepared formulations. One factor was studied at two levels and the other at three levels. The two independent factors were (A) the percentage of the gelling agent (HEC) and (B) the concentration of dried licorice roots in the prepared licorice aqueous extract. Viscosity at minimum shear stress (V_min_), pH, ulcer healing score (UHS), wound healing score (WHS), TNF-α, VEGF, EGF, and COL-1 were the computed dependent variables (Table 1).

#### 2.2.5. Characterization of the Prepared LHGs

##### pH Determination of the Prepared LHGs

The pH measurement of buccal formulations is crucial for ensuring optimal drug delivery and patient comfort. Maintaining an appropriate pH level is vital to prevent irritation or damage to the buccal mucosa and to facilitate the effective absorption of drugs through the oral mucosa [29,30]. The pH of prepared hydrogels was determined at room temperature using a Jenway pH meter (model 3510, Nottingham, UK) that was calibrated before each use with standard pH 4, 7, and 9.2 buffer solutions [31].

##### Rheological Study of the Prepared LHGs

The rheological behavior of the prepared LHGs was measured by a Brookfield cone and plate viscometer using a spindle CS-40 (DV3T, Middleboro, MA, USA). A sample volume of 3 mL was placed between the cone and plate with a configuration of 20 mm diameter/4° angle and a fixed shear rate of 1/s, operating at 25 ± 0.2 °C [32].

The rheological behavior of the formulations was studied according to Farrow’s equation [32]:

Log D = N Log S − Log η

where D, S, N, and η stand for the shear rate measured in sec-1, shear stress measured in Pa, Farrow’s constant, and viscosity measured in Pa.s, respectively. Additionally, N being the slope of the plot between log S and log D, gives a clear indication of the deviation from the Newtonian behavior. A dilatant shear-thickening flow is expected when N measures less than one value, while a shear-thinning pseudoplastic or plastic flow is foreseen when N is more than one [33].

##### Evaluation of the Biological Performance of the Prepared LHGs

*Animals*

The protocol for the present animal study was approved by the Ethics Committee of the Faculty of Oral and Dental Medicine, Future University in Egypt (No. FUE.REC (21)/9-2022). One week following acclimatization, 126 adult male Wistar rats (200–250 g) obtained from the National Research Center (Cairo, Egypt) were used in the experiment. The animals were housed in a humidity- and temperature-controlled room (60–70% and 25 ± 2 °C, respectively). Rats were fed a normal rat diet and water ad libitum prior to the dietary manipulation. The animals’ handling was adhered to the Guide for the Care and Use of Laboratory Animals (NIH publication No. 85-23, revised 1996) [34].

*Experimental Design*

**Figure 1:**
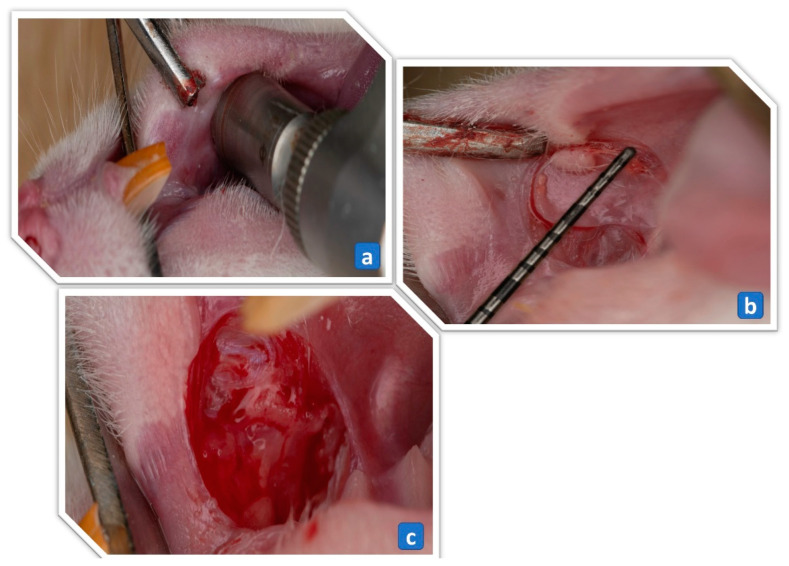
(**a**) A 5 mm diameter trephine bur mounted on a low-speed handpiece is used to incise the circular border of the ulcer. (**b**) A periodontal probe showing a 5 mm diameter of the circular incision plier. (**c**) Connective tissue floor of the ulcer following denudation of the epithelial surface with blade and tissue.

One hundred twenty-six adult male Wistar rats, weighing 200–250 g were randomly divided into 3 groups: Group A, rats treated for 3 days; Group B, rats treated for 5 days; and Group C, rats treated for 7 days. The treatment groups were further subdivided (6 rats per subgroup) into: 1. Ulcer-induced negative control (UINC) group that received no treatment (self-healing); 2. Ulcer-induced positive control (UIPC) group treated with a 2% gelling agent (LHG1); 3. UIPC group treated with a 4% gelling agent (LHG4) only; 4. UI group treated with LHG2; 5. UI group treated with LHG3; 6. UI group treated with LHG5; 7. UI group treated with LHG6. For the induction of the traumatic ulcer, rats were anesthetized with a ketamine (60 mg/kg) and xylazine (8 mg/kg) mixture through an intraperitoneal injection, and then a round trephine bur with a standard diameter (5 mm) and beveled a cutting edge (0.5 mm height) was mounted on a low-speed contra-angle handpiece. An external electric motor was used to generate handpiece rotations of 5000 rpm, with which the trephine bur was used to create an outline of a standard ulcer at the center of the right cheek mucosa (5 mm diameter and 0.5 mm depth), as shown in Figure 1a. The mucosal surface of the outlined wound area was sharply dissected using a tissue plier and a Bard Parker blade number 15 to expose the ulcer connective tissue floor as presented in Figure 1b,c [35]. The ulceration was not performed in the control group, in which the animals were anesthetized. Complete ulcer healing usually occurs in up to 14 days [36]. For that reason, the treatment regimens were chosen to be 3, 5, and 7 days to exclude the possibility of normal healing.

*Traumatic Ulcer Healing Score (UHS)*

Ulcer-induced animals in 1-UI to 7-UI groups were weighed and their ulcers were measured with a 0.5-mm precision periodontal probe on days 3, 5, and 7 to follow up with the change in ulcer surface area indicating the healing progress.

*Degree of Erythema and Exudate (DEE)*

Erythema and exudate degrees were estimated on a four-point scale ranging from 0 to 3 based upon Bhat and Sujatha [37] modifications on the Greer et al. scale [38]. The assessment was performed by three different experts, including the principal investigator, to eliminate bias as follows:

**For Erythema:**

Score 0: No erythema.

Score 1: Light red/pink.

Score 2: Red but not dark in color.

Score 3: Very red, dark in color.

**For Exudate:**

Score 0: No exudation.

Score 1: Light exudation.

Score 2: Moderate exudation.

Score 3: Heavy exudation with pseudo membrane.

*Biochemical Study*

Animals were sacrificed using a lethal dose of thiopental (IP 200 mg/kg), then the ulcer tissues were biopsied. To identify the levels of TNF-α, VEGF, EGF, and COL-1. The tissue was homogenized according to the manufacturers’ procedures and the corresponding ELISA kits were used; Rat-EGF (Epidermal Growth Factor) ELISA Kit (Elabscience Biotechnology Co., Ltd., Houston, TX, USA; Catalog #: E-EL-R0369), VEGF (Cloud-Clone Corp, TX, USA; Catalog # SEB851Ra, COL-1 (MyBioSource, Inc., San Diego, CA, USA; Catalog #: Cat No. MBS262647) and TNF-α (MyBioSource, Inc., San Diego, CA, USA; Catalog #: MBS2507393).

*Histopathological Study*

For histopathological assessment, three rats from each experimental group were sacrificed as mentioned under Section (Biochemical Study) on the third, fifth, and seventh days after ulceration. Complete-thickness samples were extracted from the mucosal tissue of the cheek and immediately fixed in 10% formalin for 48 h. After fixation, paraffin-embedded blocks were prepared and sections of 5µm thickness were obtained and stained with hematoxylin and eosin stain according to the conventional method. Slides were then assessed under a light microscope and given scores from 0 to 4 according to the following criteria [39]:

Score 0: no ulceration and remodeled connective tissue.

Score 1: no ulceration with fibrosis and slight chronic inflammation.

Score 2: ulceration with fibrosis and moderate chronic inflammation.

Score 3: ulceration with chronic inflammation process (granulation tissue).

Score 4: ulceration with acute inflammation process (dilated vessels, mixed inflammatory infiltrate with neutrophils).

#### 2.2.6. Statistical Analysis of Data

The data was displayed in the form of the mean ± SD (standard deviation). For analyzing the results of the full factorial experimental design to explore the influence of formulation inputs on the experimental outputs, Design-Expert^®^ software (version 11; Stat-Ease, Inc., Minneapolis, MN, USA) was utilized, followed by an ANOVA to test the statistical significance and separate the influence of the independent inputs. In all experiments, the statistical level of significance was set at a *p*-value ≤ 0.05. GraphPad Prism 9.1^®^ software (GraphPad Software, San Diego, CA, USA) was used to perform a one-way ANOVA; subsequently, Tukey multiple comparison tests were performed for all other statistical analyses.

## 3. Results and Discussion

### 3.1. Preparation of Licorice-Based Hydrogels (LHGs)

Exactly 500 g of licorice roots yielded 59 g of dried aqueous extract which represents 11.8%. LHGs were successfully synthesized from the aqueous extract of licorice using HEC as the natural gelling agent at room temperature. The obtained formulations showed a translucent consistency at all the examined levels, with a nearly clear glassy appearance, light, and acceptable spreadability, showing no unrecommended stiffness.

### 3.2. Identification of Metabolites in Licorice Extract Using UPLC-ESI-MS/MS

**Figure 2:**
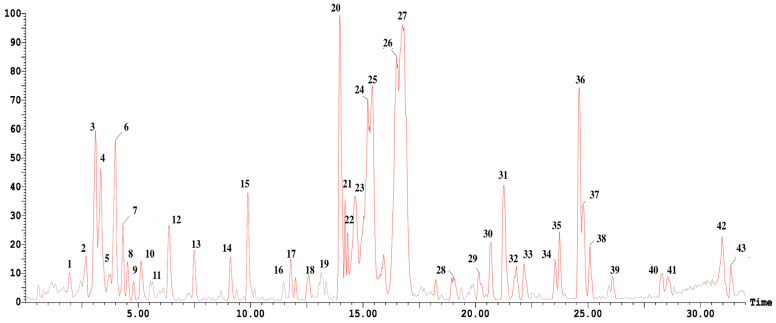
UPLC-ESI-MS/MS chromatograms in negative ion ionization mode of the aqueous extract of Licorice rhizome. The numbers on the chromatogram represent the peak numbers denoted in Table 2.

Metabolic profiling of the aqueous extract of Licorice rhizome resulted in the identification of 43 compounds representing 93.80%, mainly saponins and phenolics. Sixteen saponins were identified (58.69%) and represented mainly by Glycyrrhizic acid or Glycyrrhizin (34.85 ± 2.77%), Glycyrrhetinic acid (5.74 ± 1.02%), and Licorice saponin E2 (4.52 ± 0.98%), while 27 phenolic compounds were detected (35.11%) belonging to different classes of flavonoids and represented mainly by Isoliquiritigenin (3.64 ± 0.44%) and Isoliquiritin (3.55 ± 0.95%) as presented in Figure 2 and Table 2.

### 3.3. Statistical Analysis of the 2^1^.3^1^ Full Factorial Experimental Design

#### 3.3.1. The Influence of Formulation Factors on the pH of the Prepared LHGs

**Figure 3:**
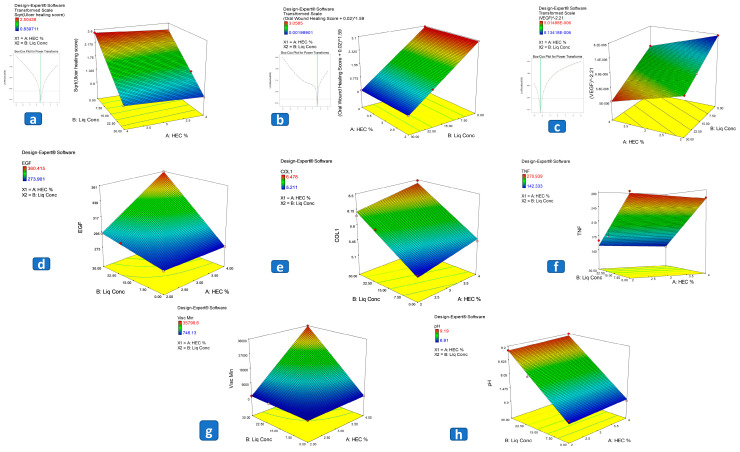
3D response surface plots for the effect of formulation factors on (**a**) ulcer healing score and its Box-Cox transformation, (**b**) wound healing score and its Box-Cox transformation, (**c**) VEGEF and its Box-Cox transformation, (**d**) EGF, (**e**) COL-1, (**f**) TNF-α, (**g**) V and (**h**) pH.

Maintaining an appropriate pH level of the buccal formulations is essential to prevent irritation or discomfort in the oral cavity. To study the level of significance of the effect of the independent factors, namely: (A) HEC concentration and (B) licorice extract concentration; on the pH of the obtained LHGs, an ANOVA test was performed. Table 1 presents the measured response, while Table 3 presents the model regression analysis. The pH values for the prepared LHGs ranged from 6.91 ± 0.02 to 9.19 ± 0.15. The ANOVA results showed that the obtained linear model exhibited a good correlation between the R^2^, predicted R^2^, and adjusted R^2^ showing values of 0.990, 0.981, and 0.923, respectively, with an adequate precision of 18.977, which ensures the reliability of the obtained model and the fact that it can be confidently used for exploring the whole design space. Results revealed that only the concentration of the dried licorice roots in the extract (B) significantly (*p* = 0.0049) increased the pH of the prepared LHGs as shown in Figure 3h. This is a logical consequence of the augmentation in the extracted amount of the basic-natured water-soluble components in licorice upon increasing the concentration of the dried herb, as Glycyrrhizin has both acidic and basic functional groups. When it dissolves in water, it can release hydroxide ions (OH-), which may contribute to an increase in pH and render the solution alkaline. This is in accordance with the findings of Tucker et al., who studied the structure of Glycyrrhizin and how it affects its performance [40].

#### 3.3.2. The Influence of Formulation Factors on the Rheological Behavior of the Synthesized LHGs

**Figure 4:**
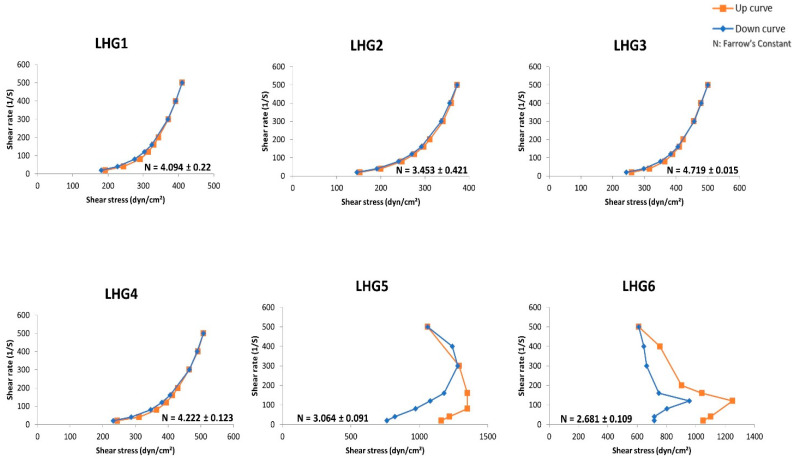
Rheograms of the prepared LHGs.

The rheological behavior of the prepared formulations represents a crucial characteristic that supports optimizing their pharmacological effect and maximizing their residence time at the site of action. The rheological behavior of the prepared LHGs was studied in terms of their viscosity at a minimum shear rate and the calculated Farrow’s constant (N), which reflects their type of flow. Although the adopted model for the analysis of N was insignificant (*p* > 0.05), all prepared LHGs exhibited an N value greater than 1, which indicates a pseudoplastic flow, as shown in Figure 4. This type of flow is preferred in pharmaceutical dosage forms generally due to the ease of application, being shear thinning, in addition to its higher viscosity at a low shear rate that allows longer residence time at the site of application. Moreover, all the obtained formulations showed a varying degree of thixotropy that is translated in the distance between the up and down curves being wider in LHG5 and LHG6 rather than LHG 1–4, which may be in favor of the former’s ability for gel-sol-gel transformation that may positively influence its residence time at the site of application.

Moreover, ANOVA results disclosed the significance (*p* = 0.0063) of the 2FI model adopted for studying the influence of the independent inputs on the viscosity at minimum shear rate (V_min_) with its nearly overlaying R^2^ and predicated R^2^ (1.000 and 0.999, respectively) and the extremely high adequate precession of 252.135, which augments the reliability of the model to investigate the whole design space confidently, as shown in Table 3. The values for V_min_ are presented in Table 1, and the regression results are shown in Table 3. The results revealed the significance of all the independent factors and the factor interactions (*p* = 0.0052, *p* = 0.0056, and *p* = 0.0057 for A, B, and AB, respectively). The increase in HEC concentration (A) with the increase in dried Licorice roots concentration (B) significantly (*p* = 0.0057) increased V_min_, as shown in Figure 3g. This is surely attributed to the gelling effect of HEC, which creates a gel-sol effect or, in other words, a thixotropic effect that increases the viscosity at a minimum shear rate. Moreover, Glycyrrhizin, whose concentration is expected to be higher with increasing the concentration of the dried licorice roots in the prepared hydrosol, has a proven gelling effect that is nowadays being considered as a green alternative to synthetic gelling agents such as carbopols. All of which synergistically increased the values of V_min_. This is in accordance with the findings of Tucker et al., who studied the self-assembly and gelation properties of Glycyrrhizin [40].

#### 3.3.3. The Influence of Formulation Factors on the Traumatic Ulcer Healing Score (UHS)

Among different natural phytochemicals, licorice extracts demonstrated a significant impact in the management of a wide array of oral diseases such as dental caries, candidiasis, gingivitis, periodontitis, oral cancer, and recurrent aphthous ulcers. Licorice root possesses several bioactive metabolites that provide a strong anti-inflammatory and anti-oxidant effect, such as glabridin, licoricidin, licorisoflavan A, licochalcone A, and glycyrrhizin [12]. Despite abundant evidence that supports the favorable outcomes of using licorice in treating oral diseases, differences between different concentrations and delivery forms regarding the healing of traumatic ulcers have not been investigated.

Successful wound healing relies on a rapid, uncomplicated wound closure. This is judged by a careful assessment of wound margin contraction towards the center [41].

The linear model used to investigate the influence of the independent inputs on the measured UHS proved significant yet needed fine tuning upon applying the Box-Cox diagnostic test, which searches for the best lambda to augment the biased variables, thus creating a best-fitting model equation for the data [42,43]. The existing lambda was overlaid with the optimum lambda (0.5) for the selected model to augment its reliability, as shown in Figure 3a and Table 3. ANOVA results revealed a good correlation between R^2^, predicted R^2^, and adjusted R^2^ (0.999, 0.998, and 0.997, respectively) with an adequate precision of 67.486, which assures the capability of the model to predict the experiments that were not conducted.

ANOVA results revealed the significance (*p* = 0.008) of the adopted model with the independent variable B as the only significant variable (*p* = 0.0004). The two concentrations of dried Licorice roots used 20% and 30%, corresponding to formulations LHG2, LHG3, LHG5, and LHG6, showed statistically significant favorable wound healing index outcomes in comparison to the control groups. This was in line with Assar et al., who reported a statistically significant wound healing acceleration by the increase in collagen deposition and angiogenesis when licorice supplementation was administered to Wister rat skin wounds. The improved cutaneous wound healing in the test group of the latter study was justified by the free radical scavenging potential, potent anti-inflammatory, and antioxidant activities of the licorice [11]. Similarly, Zaki et al. reported a faster wound contraction in rabbits treated with topical licorice compared to the negative control and animals treated with topical application of the vehicle Eucerin [44]. The results of the current study coincide with the clinical trial of Raeesi et al., who reported a statistically significant reduction in the wound size and necrotic zone on the third and fifth days following the application of 5% licorice bioadhesive paste to recurrent aphthous ulcers in comparison to the control palliative therapy and plain bioadhesive paste [45]. Burgess et al. investigated the effect of using licorice extract in the form of a dissolving oral patch that was applied to recurrent aphthous ulcers in a randomized clinical trial. In comparison to a negative control group, the test group demonstrated faster ulcer healing and significantly lower passive and stimulated pain which correspond with the results of the present study [46].

On the third day post-ulceration (Group A), higher concentrations of the licorice significantly yielded superior outcomes in terms of erythema, where no erythema (score 0) was found in 50% of the rats while a light pink color (score 1) was observed in the other 50% in group LHG6. This percentage was increased on day 5 post ulceration (Group B) to reach 83.33% with no erythema (score 0) while only 16.66% showed light red color (score 1). On day 7 post ulceration (Group C) all the ulcers in 100% of the rats in group LHG6 showed no erythema (score 0). Unlike group LHG6, group UINC showed very dark red erythema (score 3) in 83.33% of the rats in the group and red erythema (score 2) in the remaining 16.66% on the third day post ulceration (Group A). The results improved on day 5 (Group B) to reach 66.66% of the rats with a score of 2 and 33.33% with a score of 1. On the seventh day (Group C), although the results continued to improve to reach 83.33% of the rats showing light erythema (score 1) and only 16.66% with dark red erythematous ulcer (score 2), none of the studied rats in UINC group reached score 0.

Regarding the exudation, all the ulcers (100%) in groups LHG5 and LHG6 showed no exudation (score 0) on day 7 post-ulceration. On the contrary, in the UINC group, 66.66% of the rats showed light exudation (score 1) while 33.33% showed moderate exudation (score 2) on the seventh day post ulceration.

In conclusion, higher concentrations of the licorice yielded superior outcomes in terms of degree of erythema and exudation compared to the self-healing group, as shown in Table 4 and Table 5.

#### 3.3.4. The Influence of Formulation Factors on the Investigated Biological Markers

**Figure 5:**
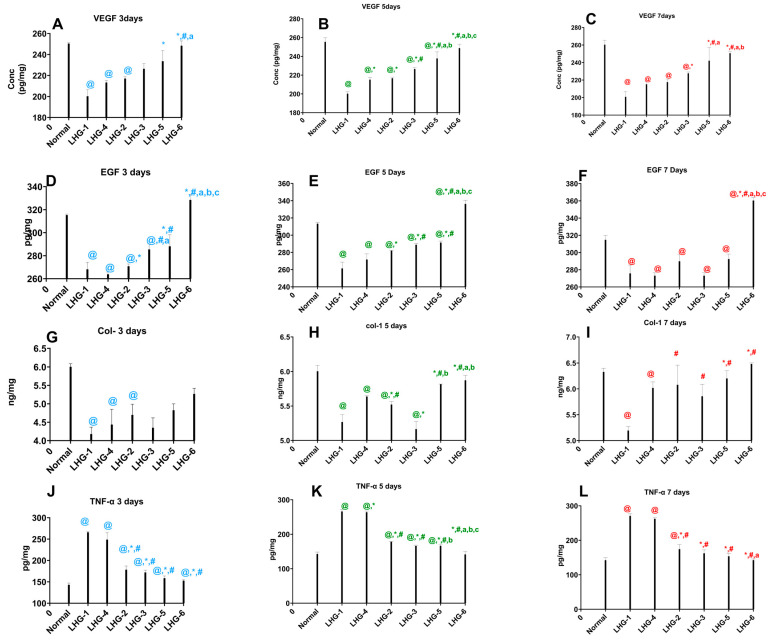
The effect of formulas (LHG-1-LHG-6) on the levels of: (**A**–**C**) Tissue VEGF during the 3, 5, and 7 days of treatment; respectively, (**D**–**F**) Tissue EGF during the 3, 5, and 7 days of treatment; respectively, (**G**–**I**) Tissue COL-1 during the 3, 5, and 7 days of treatment; respectively, and (**J**–**L**) Tissue TNF-α during the 3, 5, and 7 days of treatment; respectively. Values are means ± SD. Statistical analysis was carried out using ANOVA followed by Tukey’s post hoc test, with *p*-values at 7 days: (VEGF, EGF, TNF-α: *p* < 0.0001 and COL-1: <0.0011). F-values at 7 days: (COL-1: 7.305 EGF: 49.63, VEGF: 29.9 TNF-α: 147.1) As compared with (@) Normal group, (*) LHG-1 group, (#) LHG-4 group, (a) LHG-2 group, (b) LHG-3 group, and (c) LHG-5 group in the same time interval.

A linear model was constructed to study the effect of the independent factors on the expression of COL-1 measured in the 7-day group (Group C). ANOVA results revealed the significance (*p* = 0.0199) of the adopted model, with no need for further transformation, translated in the close correlation between its R^2^, predicted R^2^, and adjusted R^2^ (0.9801, 0.9602, and 0.8410, respectively) and the adequate precision value exceeding 4 which indicates the reliability of the model to predict the whole design, including the un-carried experiments. The measured response values are shown in Table 1, while the results of the regression analysis are presented in Table 3. Only the concentration of dried licorice roots had a significant (*p* = 0.0109) effect on the increase in expression of COL-1 throughout the treatment period, as shown in Figure 3e and Figure 5G–I.

In the first 3 days of treatment, the decline in the levels of COL-1 due to the traumatic ulcer was not amended by all the treatment groups. After 5 days of the treatment, the LHG5-treated group and LHG6-treated group showed comparable COL-1 levels to the normal tissues. On the other hand, the other LHG2 and LHG3 treated groups showed a significant enhancement to the COL-1 levels in the tissues compared to the LHG1 and LHG4 treated groups, prepared only with the gelling agent with concentrations of 2% and 4%, respectively. The increase in the percentage of dried licorice roots to 30% in LHG3 showed a 3.16% significant increase in the COL-1 levels in the epithelial tissues compared to the 20% LHG2 at the lower level of HEC (2%). In addition to that, it was noticed that the group treated with LHG6 formula prepared using the 30% concentration of dried licorice roots showed a significant increase in COL-1 levels by 4.4% compared to the group treated with LHG2 formula containing 20% licorice (*p* ≤ 0.0001, F = 53.31). The highest levels of tissue collagen were found after the 7 days treatment duration where 27.8% enhancement in COL-1 levels was noticed between collagen levels in the 3 days interval treatment compared to the 7 days interval regimen with LHG3 formula. By the same token, a notable 23% increase in COL-1 levels was noticed between collagen levels in the 3 days interval treatment compared to the 7 days interval regimen upon the treatment with LHG6 formula (*p* ≤ 0.0011, F = 7.305).

Additionally, to determine the level of significance of the independent factors on the level of EGF in the 7-days treatment group (Group C), an ANOVA test was conducted. The measured responses are presented in Table 1 and the regression results are shown in Table 3. The created 2FI model proved its significance (*p* = 0.0394) with a very high correlation between the R^2^ and the predicted R^2^ (0.9990 and 0.9962, respectively) and a favorably high adequate precision of 43.899, all of which augment the reliability of the constructed model. Results revealed the significance of the two independent factors and their interaction (*p* = 0.0401, *p* = 0.0267, and *p* = 0.0403 for A, B, and AB, respectively) on the values of EGF, as shown in Table 3 and Figure 3d and Figure 5D–F.

On the third day of treatment, only the LHG6-treated group normalized the levels of EGF, and the pattern continued in the following days where the treatment with that formula showed a significant increment in the levels of EGF compared to the normal group (*p* ≤ 0.0001, F = 66.7). After the 5 days treatment interval, the LHG3, LHG5, and LHG6 treated groups showed a significant increase in the EGF levels by 10.5%, 11.4%, and 28.6%, respectively, compared to the LHG1 positive control group. In addition to that, the higher concentrations of HEC in the LHG5- and LHG6-treated groups showed a significant incline in the EGF levels by 3.3% and 19.2% compared to the LHG2-treated group. Moreover, it was observed that the increase in the dried licorice root concentration in the LHG6 formula showed a 15.41% significant increase in the levels of EGF compared to the LHG5-treated group. Similarly, the LHG6-treated group showed the highest increment in the EGF levels by 23.24% compared to that in the LHG5-treated group, which shed light on the effect of licorice on the assessed growth factor (*p* ≤ 0.0001, F = 110.3). Accordingly, it was discerned that as the treatment continued for 7 days the highest levels of EGF were found upon treatment with the LHG6 formula, which showed a significant increment in EGF levels by 31% and 32% compared to LHG1 and LHG4 treated groups, respectively, as displayed in Figure 3g and Figure 5D–F (*p* ≤ 0.0001, F = 49.63).

To investigate the significant effect of the formulation factors on the level of VEGF after treatment for 7 days, a linear model was constructed as shown in Table 3. The model proved significant but required further transformation upon applying the Box-Cox analysis to ensure the reliability of the model to investigate the whole design space. The current lambda was super-imposed with the best lambda (−2.21) to improve the skewed variables and create a model equation that best fits the data highlighted with the values of the R^2^, predicted R^2^, and adjusted R^2^ being 1 and adequate precision of 762.604. The values of the measured responses are presented in Table 3. ANOVA results revealed the significance of both HEC and dried Licorice root concentration in augmenting the level of VEGF, as shown in Figure 3c and Figure 5A–C.

Upon treatment for 3 days, LHG5 and LHG6 formulas showed a significant increase in the level of VEGF compared to the LHG1 (2% HEC only) treated group by 16.6% and 23.9%, respectively (*p* ≤ 0.0001, F = 10.87). Similarly, the formulas LHG5 and LHG6 showed a significant increase compared to the LHG2 treated group by 18.7% and 24.2%, respectively, as presented in Figure 5A–C (*p* ≤ 0.0001, F = 81.73). Likewise, after the 7 days treatment interval, the LHG6 formula showed the most profound increase in the levels of VEGF significantly by 25% compared to the LHG1 group. In fact, it was noteworthy that the levels of VEGF increased notably towards normalization upon treatment with LHG6 formula during all assessed treatment intervals (*p* ≤ 0.0001, F = 29.9).

Finally, to study the effect of the independent factors on the level of TNF-α after 7 days of treatment. ANOVA results revealed the significance (*p* = 0.0279) of the constructed linear model with B (dried Licorice roots concentration) as the only significant (*p* = 0.0141) factor. The validity of the model was evaluated by comparing the closeness of the values of R^2^, predicted R^2^, and adjusted R^2^ (0.97, 0.94, and 0.90, respectively) and the value of the adequate precision being higher than 4 (11.29), which assures that the model can be used to study the whole design space. Measured values are presented in Table 1 while the regression results are shown in Table 3 and Figure 3f and 5J–L.

Upon the measurement of the TNF-α in the tissues, during the 3 days period, the levels of TNF-α were found to be the highest in the two groups receiving treatment with LHG1 and LHG4 (2% and 4% gelling agent only, respectively) (*p* ≤ 0.0001, F = 108.2). On the 5 days treatment groups, only the LHG6-treated group showed significant normalization of the levels of TNF-α compared to the control group in addition to a significant decrease in the levels of the proinflammatory TNF-α by 25.9% and 17.3% compared to the LHG2- and LHG3-treated groups, respectively (*p* ≤ 0.0001, F = 333.3). Upon the treatment for 7 days, the same pattern was attained in addition to the success of the LHG3 and the LHG5 formulas to significantly normalize the levels of TNF-α. In fact, all treatment formulas showed notable differences in decreasing the levels of TNF-α after 7 days of treatment with the most incline reserved for the LHG6-treated group (4% HEC and 30% Licorice) by 88% and 86.5% compared to the LHG1-(2% gelling agent only) and LHG4-treated groups, as shown in Figure 5J–L (*p* ≤ 0.0001, F = 147.1).

Traumatic ulcer healing requires plentiful finely tuned procedures that occur in a specific sequence an important part of this sequence is collagen-1 (COL-1) which plays a central role in the wound healing process, actively participating in the complex mechanisms of tissue mending and rejuvenation. As the predominant protein within the extracellular matrix of connective tissues, collagen-1 bestows essential structural stability and reinforcement to the wounded area. This, in turn, expedites the creation of fresh tissue and the eventual sealing of the wound [6,47]. The highest significant results were found in the formulas with the hydrosol prepared using 30% dried licorice roots, i.e., LHG3 and LHG6 during the 7 days of treatment. This is in line with a previously published work suggesting the effect of their treatments to be apparent on the levels of COL-1 in the same time interval [48]. As the wound progresses to the proliferative phase, fibroblasts synthesize collagen-1 to form a network of fibers that enhance tissue strength and promote angiogenesis [49]. Upon the assessment of VEGF in the tissues, the treatment after 7 days showed the highest levels of VEGF which significantly showed increased levels of VEGF compared to the normal control and the positive control with the gelling agents (2% and 4%), the effect of VEGF on the acceleration of wound healing was highlighted in previous studies [50]. Moreover, another recent study suggested the importance of licorice extract in wound healing owing to its antioxidant capacity [11]. Moreover, the effect of licorice-based therapy was explored on the levels of epithelial growth factor (EGF) in the ulcer tissue, which activates the secretion of COL-1 that ensures that the healed tissue acquires optimal strength and flexibility. It was observed that the highest licorice concentration showed the largest increment in the amount of EGF in all the treatment periods. Which suggests the greatest ulcer healing capacity. Within hours of the injury, the body stimulates the release of the EGF to stimulate cell migration and proliferation [51]. TNF-α is an inflammatory cytokine that can inhibit angiogenesis in diabetic mice, and its inhibition was found to enhance wound healing [52]. That conclusion agrees with the conclusion of the current study which showed that the licorice extract declined the TNF-α levels expressed within the ulcer tissues which results in faster ulcer healing.

In conclusion, the treatment with the 30% licorice extract and 4% HEC formulation (LHG6) for 7 days showed a significant increase in the growth factors (EGF and VEGF) which in turn activates the increase of collagen (COL-1). On the other hand, it showed a significant decrease in the pro-inflammatory TNF-α. This suggests a better healing capability of that formula compared to all other treatment groups.

#### 3.3.5. The Influence of Formulation Factors on Wound Healing Score (WHS)

Wound healing is a dynamic process of accurately programmed phases including inflammation, cell proliferation, wound contraction, angiogenesis, matrix remodeling, and epithelialization, a process designed to restore the damaged tissue to its original status. Initially, the inflammatory phase that follows tissue damage shows the domination of neutrophils to carry out phagocytosis, later modification of the inflammatory cellular infiltration occurs and lymphoplasmacytic infiltration dominates, which is a distinctive feature of chronic inflammation. This is followed by the proliferative phase, which includes epithelialization, angiogenesis, and dominance of fibroblasts. Finally, collagen production and remodeling follow to ultimately end in wound healing [39,53,54,55].

To study the effect of the formulation factors on the WHS, a Linear model was constructed that required further transformation upon diagnosis using Box-Cox. Therefore, the current lambda was superimposed with the best lambda (−1.29) to ensure the best fit of the created equation to the data as shown in Figure 3b. The measured values are shown in Table 1 while the regression data is presented in Table 3. ANOVA results revealed the high significance (*p* < 0.0001) of the constructed model with R^2^, predicted R^2^, and adjusted R^2^ values of 1, and adequate precision of 618.518 which ensures the reliability of the model. Results showed that only factor B (dried Licorice roots concentration) had a significant (*p* < 0.0001) effect on the measured WHS.

**Figure 6:**
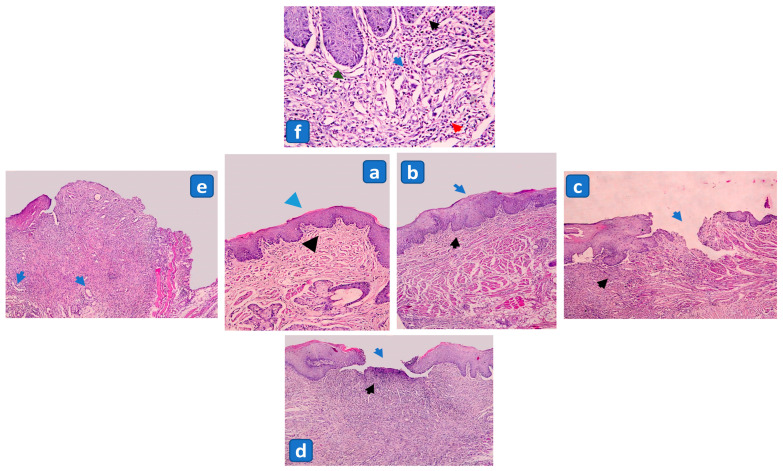
Photomicrograph of rat buccal mucosa showing: (**a**) intact epithelium with no ulceration (blue arrow) and remodeled connective tissue (black arrow) (score 0) (H&E; ×100); (**b**) normal epithelium without ulceration (blue arrow) and slight chronic inflammation (black arrow) (score 1) (H&E; ×100); (**c**) discontinuity of epithelium (blue arrow) along with fibrosis and moderate chronic inflammation (black arrow) (score 2) (H&E; ×100); (**d**) ulceration (blue arrow) and granulation tissue with chronic inflammatory process (black arrow) (score 3) (H&E; ×100); (**e**) ulceration area that displays dilated blood vessels (blue arrows) and mixed inflammatory infiltrate (score 4) (H&E; ×100); (**f**) mixed inflammatory cells including eosinophils (blue arrow), neutrophils (black arrow), plasma cells (red arrow), and lymphocytes (green arrow) (score 4) (H&E; ×400).

In Group A, day three post-ulceration investigations were carried out where in groups treated with LHG1, LHG4, LHG3, and LHG5, 66.67% of the rats revealed ulceration with formation of granulation tissue and chronic inflammation process (score 3), as shown in Figure 6d. Only 33.33% of the rats revealed discontinuity of epithelium, along with acute inflammation process, dilated vessels, and mixed inflammatory infiltrate with neutrophils (score 4) as illustrated in Figure 6e,f. Meanwhile, in the group treated with LHG2, 66.67% of the rats showed discontinuity of epithelium, along with acute inflammation process, dilated vessels, and mixed inflammatory infiltrate with neutrophils (score 4). In addition, 33.33% demonstrated ulceration with formation of granulation tissue and chronic inflammation process (score 3). In the group treated with LHG6, 66.67% of the rats exhibited ulceration with fibrosis and moderate chronic inflammation (score 2) as shown in Figure 6c, while 33.33% showed ulceration with formation of granulation tissue and chronic inflammation process (score 3). Lastly, in the UINC group, all rats revealed discontinuity of epithelium, along with acute inflammation process, dilated vessels, and mixed inflammatory infiltrate with neutrophils (score 4), as shown in Table 6.

In Group B, day five post-ulceration investigations were carried out where in the LHG1 treatment group, 66.67% of the rats displayed ulceration with fibrosis and moderate chronic inflammation (score 2), while 33.33% showed ulceration with formation of granulation tissue and chronic inflammation process (score 3). In LHG2 and LHG4 treated groups, 33.33% demonstrated no ulceration along with fibrosis and slight chronic inflammation (score 1) and 66.67% showed ulceration with fibrosis and moderate chronic inflammation (score 2) as illustrated in Figure 6b,c, respectively. In the LHG3-treated group, 66.67% of the rats demonstrated no ulceration along with fibrosis and slight chronic inflammation (score 1), while 33.33% showed ulceration with fibrosis and moderate chronic inflammation (score 2). Meanwhile, in the LHG5-treated group, all rats (100%) showed no ulceration along with fibrosis and slight chronic inflammation (score 1). In addition, in the LHG6-treated group, 66.67% demonstrated no ulceration and re-modeled connective tissue (score 0) and 33.33% of the rats showed no ulceration along with fibrosis and slight chronic inflammation (score 1) as shown in Figure 6a,b. In the UINC group, 33.33% of the rats showed ulceration with fibrosis and moderate chronic inflammation (score 2), while 66.67% demonstrated ulceration with the formation of granulation tissue and chronic inflammation processes (score 3).

Finally in Group C, seven days post-ulceration the investigations showed that in the LHG1-treated group, 66.67% of the rats demonstrated no ulceration along with fibrosis and slight chronic inflammation (score 1), while 33.33% showed ulceration with fibrosis and moderate chronic inflammation (score 2). In LHG2- and LHG4-treated groups, 33.33% showed no ulceration and remodeled connective tissue (score 0), 33.33% demonstrated no ulceration along with fibrosis and slight chronic inflammation (score 1), and 33.33% showed ulceration with fibrosis and moderate chronic inflammation (score 2). In LHG3- and LHG6-treated groups, 66.67% of the rats demonstrated no ulceration and remodeled connective tissue (score 0), and 33.33% showed no ulceration along with fibrosis and slight chronic inflammation (score 1). In the group treated with LHG5, 33.33% of the rats showed no ulceration and remodeled connective tissue (score 0), and 66.67% displayed no ulceration along with fibrosis and slight chronic inflammation (score 1). Finally, in the self-healing control group, 33.33% demonstrated no ulceration along with fibrosis and slight chronic inflammation (score 1), while 66.67% showed ulceration with fibrosis and moderate chronic inflammation (score 2), as summarized in Table 6.

These obtained results revealed that, when topically applied, Licorice significantly enhanced the healing of oral mucosal ulcers and reduced the re-epithelialization period in comparison to control groups. In accordance with our histological results, Assar et al. reported accelerated cutaneous wound healing in Licorice-treated rats with complete re-epithelialization and maximum maturation of granulation tissue with well-organized accumulation of collagen fibers in the dermis of the wounded area [11]. Similarly, Oloumi et al. examined the effect of Licorice root extract on dermal wounds in rats and the study revealed an increase in the number of fibroblasts, in addition to better re-epithelialization in Licorice-treated groups when compared to control groups [56]. Moreover, Hanafi et al. also documented that Licorice creams significantly improved wound healing in Guinea pigs [57]. Furthermore, Najeeb and Al-Refai reported that Licorice root extract can shorten the healing time of induced oral mucosal wounds in rabbits [58].

## 4. Conclusions

In conclusion, the green development of licorice-based hydrogels (LHGs) using hydroxyethyl cellulose (HEC) as a natural gelling agent has shown significant potential for enhancing the healing of oral mucosal ulcers. The study systematically investigated the effects of dried licorice roots concentration and HEC concentration on various properties of the hydrogels, including pH, rheological behavior, and their impact on wound healing parameters such as traumatic ulcer healing score (UHS), collagen-1 expression (COL-1), growth factors (EGF, VEGF), and pro-inflammatory marker (TNF-α). The results demonstrated that LHGs with higher concentrations of licorice extract (30%) and HEC (4%) exhibited improved wound healing outcomes, including accelerated re-epithelialization, increased collagen production (27.8%), elevated growth factor expression (23.24%), and reduced inflammation (88%). These findings underscore the therapeutic potential of the developed licorice-based hydrogels in the sustainable addressing of oral ulcers, offering a promising avenue for the development of effective and natural wound-healing solutions.

## Figures and Tables

**Table 1:** Dependent and independent variables of the 2^1^.3^1^ full factorial experimental design of the Licorice-based hydrogels (LHGs).

Formula	Independent Factors		Dependent Factors *						
	HEC g% (*w*/*w*)	Dried Licorice Roots Concentration g% (*w*/*w*)	pH	V_min_ (cp)	UHS	COL-1 (ng/mg)	EGF (pg/mg)	VEGF (pg/mg)	TNF-α (pg/mg)
LHG1	2	0	7.03 ± 0.01	819.16 ± 10.93	5.61 ± 0.12	5.21 ± 0.08	275.80 ± 9.64	200.92 ± 6.13	270.94 ± 6.13
LHG2	2	20	8.19 ± 0.06	746.13 ± 15.13	2.09 ± 0.09	5.85 ± 0.38	289.85 ± 8.29	217.67 ± 3.47	174.00 ± 13.12
LHG3	2	30	9.03 ± 0.05	1000.14 ± 29.40	0.82 ± 0.02	6.01 ± 0.23	292.75 ± 11.64	227.71 ± 1.51	162.44 ± 9.88
LHG4	4	0	6.91 ± 0.02	1016.01 ± 13.01	6.27 ± 0.12	5.43 ± 0.12	273.90 ± 3.38	215.23 ± 2.87	262.11 ± 3.24
LHG5	4	20	8.01 ± 0.05	2127.28 ± 72.84	0.71 ± 0.01	6.28 ± 0.15	293.53 ± 5.81	242.10 ± 15.16	153.54 ± 8.02
LHG6	4	30	9.19 ± 0.15	35798.6 ± 121.01	1.08 ± 0.08	6.48 ± 0.02	360.41 ± 3.52	250.08 ± 2.10	142.33 ± 3.30

* *n* = 3, All values = mean ± SD, *p*-value: (EGF, VEGF and TNF-α)< 0.0001 and COL-1: <0.0011) and F-values: (COL-1: 7.305 EGF: 49.63, VEGF: 29.9 TNF-α: 147.1); HEC: Hydroxyethylcellulose, V_min_: Viscosity at minimum shear rate, UHS: Ulcer healing score, COL-1: Collagen type 1, EGF: Epithelial growth factor, VEGF: Vascular endothelial growth factor and TNF-α: inflammatory cytokine.

**Table 2:** UPLC-ESI-MS/MS in negative ion ionization mode of the aqueous extract Licorice rhizome.

Peak No.	Rt	Compound	Class	Relative Abundance (%)	Molecular Formula	[M−H]- (*m*/*z*)	MS2 Fragments
1	2.41	Neoliquiritin	Phenolic	2.46 ± 0.23	C_21_H_21_O_9_	418.134	257,239,137
2	3.16	Glycyrol	Phenolic	1.44 ± 0.26	C_21_H_17_O_6_	365.110	257,147
3	3.53	Glycyrrhetol	Saponin	0.78 ± 0.17	C_30_H_47_O_3_	455.360	452,137
4	3.71	Glabric acid	Saponin	2.11 ± 1.02	C_30_H_45_O_5_	486.345	469,451,317
5	4.23	Isoliquiritigenin	Phenolic	3.64 ± 0.44	C_15_H_11_O_4_	255.065	255,135
6	4.31	Isoliquiritin	Phenolic	3.55 ± 0.95	C_21_H_21_O_9_	417.119	255,135
7	4.58	Licorice saponin G2	Saponin	0.49 ± 0.07	C_42_H_61_O_17_	837.386	351,289
8	4.89	Neoisoliquiritin	Phenolic	2.12 ± 0.55	C_21_H_21_O_9_	417.119	257,147
9	5.32	Dehydroglyasperin D	Saponin	0.92 ± 0.09	C_22_H_23_O_5_	368.112	298,162
10	5.50	Glucoliquiritin apioside	Saponin	1.07 ± 0.14	C_32_H_39_O_18_	711.121	256
11	5.73	Glycyrrhetinic acid	Saponin	5.74 ± 1.02	C_30_H_45_O_4_	470.347	452,406
12	6.26	Licoflavone B	Phenolic	0.25 ± 0.08	C_25_H_25_O_4_	389.174	333
13	7.44	Licochalcone D	Phenolic	0.94 ± 0.06	C_21_H_21_O_5_	353.141	338,297
14	8.98	Licorice saponin A3	Saponin	0.59 ± 0.11	C_48_H_71_O_21_	1000.446	825,649,451
15	10.25	Neolicuroside	Phenolic	1.54 ± 0.21	C_26_H_29_O_13_	549.160	255,135
16	11.42	Glabrolide	Saponin	0.79 ± 0.24	C_30_H_43_O_4_	468.331	451,439,395
17	11.68	Nicotiflorin	Phenolic	1.04 ± 0.09	C_27_H_29_O_15_	593.576	461,414,374
18	12.50	Licochalcon B	Phenolic	0.77 ± 0.55	C_16_H_13_O_5_	385.078	270
19	13.01	Licorice saponin J2	Saponin	0.28 ± 0.13	C_42_H_63_O_16_	824.236	454,436,314
20	13.76	Glycyrrhizic acid (Glycyrrhizin)	Saponin	34.85 ± 2.77	C_42_H_61_O_16_	821.396	351,193
21	13.92	Glycyrrhetic acid	Saponin	2.60 ± 0.95	C_30_H_45_O_4_	469.330	451,317
22	14.24	3-hydroxyglabrol	Phenolic	1.36 ± 1.01	C_25_H_27_O_5_	407.185	198
23	14.88	Liquoric acid	Saponin	0.52 ± 0.04	C_30_H_43_O_5_	483.318	450,193
24	15.05	Isoglabrolide	Saponin	0.23 ± 0.03	C_30_H_43_O_4_	468.331	451,439,395
25	15.34	Licoflavonol	Phenolic	0.72 ± 0.15	C_20_H_17_O_6_	353.401	135
26	16.41	Licorisoflavan A	Phenolic	0.58 ± 0.12	C_27_H_33_O_5_	437.231	167,135
27	16.52	Glucoisoliquiritin	Phenolic	0.11 ± 0.04	C_27_H_30_O_14_	579.169	417,255
28	18.29	Glabrene	Phenolic	0.76 ± 0.17	C_20_H_17_O_4_	321.113	277
29	20.11	Liquiritin apioside	Phenolic	1.25 ± 0.05	C_26_H_29_O_13_	549.155	429,255,135
30	20.66	Isoglycyrol	Phenolic	0.26 ± 0.11	C_21_H_17_O_6_	366.233	335,321,203
31	21.18	Glucoliquiritin	Phenolic	0.53 ± 0.05	C_27_H_31_O_14_	579.169	417,255
32	21.79	Hispaglabridin	Phenolic	0.72 ± 0.02	C_25_H_27_O_4_	391.438	215,177
33	22.05	Glabridin	Phenolic	2.46 ± 0.23	C_20_H_19_O_4_	323.127	305,201,135
34	23.43	Licorice saponin E2	Saponin	4.52 ± 0.98	C_42_H_59_O_16_	819.383	383,352
35	23.52	Glabrol	Phenolic	1.14 ± 0.07	C_25_H_27_O_4_	391.189	221,203,187
36	24.22	Isoviolanthin	Phenolic	1.03 ± 0.12	C_27_H_29_O_14_	577.149	559,503,415
37	24.61	Formononetin	Phenolic	0.39 ± 0.02	C_16_H_11_O_4_	268.014	253,237,137
38	24.98	Glyzaglabrin	Phenolic	0.86 ± 0.03	C_16_H_9_O_6_	297.250	135
39	26.03	Liquiritigenin	Phenolic	1.60 ± 0.45	C_15_H_11_O_4_	247.081	165,137
40	28.39	Liquiritin	Phenolic	2.47 ± 0.23	C_21_H_21_O_9_	417.155	255,135
41	28.46	Liquiritin apioside	Phenolic	1.12 ± 0.03	C_26_H_29_O_13_	550.177	257,239,137
42	30.77	Glyasperin D	Saponin	1.09 ± 0.77	C_22_H_25_O_5_	370.103	249,218,204
43	31.32	Licorice saponin K2	Saponin	2.11 ± 0.65	C_42_H_61_O_16_	821.395	351
Total no. of identified compounds				43			
Total % of identified compounds				93.80			
Total no. of identified saponins				16			
Total % of identified saponins				58.69			
Total no. of identified phenolics				27			
Total % of identified phenolics				35.11			

**Table 3:** Model parameters of the 2^1^.3^1^ full factorial experimental design of the prepared LHGs.

ANOVA	pH	V_min_	UHS	COL-1	EGF	VEGF	TNF-α	WHS
Model	Linear	2FI	Liner	Liner	2FI	Linear	Linear	Linear
R-Squared	0.990	1.000	0.999	0.98	1.00	1.00	0.97	1.00
Adj R-Squared	0.981	0.999	0.998	0.96	1.00	1.00	0.94	1.00
Pred R-Squared	0.923	N/A	0.997	0.84	N/A	1.00	0.90	1.00
Adeq Precision	18.977	252.135	67.486	16.25	43.90	762.60	11.29	618.518
Coded Equation	pH = +8.01 + 0.042 * A + 1.06 * B	Visc Min = +9627.35 + 8779.96 * A + 8730.52 * B + 8660.78 * A * B	Sqrt(Ulcer healing score) = +1.71 + 0.059 * A − 0.73 * B	COL1 = +5.80 + 0.16 * A + 0.47 * B	EGF = +301.16 + 16.00 * A + 26.01 * B + 17.24 * A * B	(VEGF)^−2.21 = +6.575 × 10^−6^ −5.740 × 10^−7^ * A −9.847 × 10^−7^ * B	TNF = +205.58 −3.36 * A −58.36 * B	(WHS + 0.02)^1.59 = +1.53 − 1.826 × 10^−3^ * A − 1.53 * B

V_min_: Viscosity at minimum shear rate, UHS: Ulcer healing score, COL-1: Collagen type 1, EGF: Epithelial growth factor, VEGF: Vascular endothelial growth factor and TNF-α: inflammatory cytokine.

**Table 4:** The degree of erythema in the different studied groups on days 3, 5, and 7 *.

Day	Score	Experimental Groups (Number of Rats)						
		1-UINC Self-Healing	2-UIPC LHG1	3-UIPC LHG4	4-UI LHG2	5-UI LHG3	6-UI LHG5	7-UI LHG6
Day 3	Score 0	NF	NF	NF	NF	NF	NF	3
	Score 1	NF	NF	NF	1	NF	5	3
	Score 2	1	3	3	5	3	1	NF
	Score 3	5	3	3	NF	3	NF	NF
	Score 4	NF	NF	NF	NF	NF	NF	NF
Day 5	Score 0	NF	NF	NF	NF	2	4	5
	Score 1	2	NF	2	5	4	2	1
	Score 2	4	6	4	1	NF	NF	NF
	Score 3	NF	NF	NF	NF	NF	NF	NF
	Score 4	NF	NF	NF	NF	NF	NF	NF
Day 7	Score 0	NF	NF	2	3	4	5	6
	Score 1	5	3	3	3	2	1	NF
	Score 2	1	3	1	NF	NF	NF	NF
	Score 3	NF	NF	NF	NF	NF	NF	NF
	Score 4	NF	NF	NF	NF	NF	NF	NF

* NF: Not Found, *n* = 6 in each group.

**Table 5:** The degree of exudation in the different studied groups on days 3, 5, and 7 *.

Day	Score	Experimental Groups (Exudate) (Number of Rats)						
		1-UINC Self-Healing	2-UIPC LHG1	3-UIPC LHG4	4-UI LHG2	5-UI LHG3	6-UI LHG5	7-UI LHG6
Day 3	Score 0	NF	NF	NF	NF	NF	1	4
	Score 1	NF	NF	NF	1	3	4	2
	Score 2	1	3	4	4	2	1	NF
	Score 3	3	3	2	1	1	NF	NF
	Score 4	2	NF	NF	NF	NF	NF	NF
Day 5	Score 0	NF	NF	NF	NF	3	4	5
	Score 1	2	1	3	5	3	2	1
	Score 2	4	5	3	1	NF	NF	NF
	Score 3	NF	NF	NF	NF	NF	NF	NF
	Score 4	NF	NF	NF	NF	NF	NF	NF
Day 7	Score 0	NF	NF	1	4	5	6	6
	Score 1	4	5	3	2	1	NF	NF
	Score 2	2	1	2	NF	NF	NF	NF
	Score 3	NF	NF	NF	NF	NF	NF	NF
	Score 4	NF	NF	NF	NF	NF	NF	NF

* NF: Not Found, *n* = 6 in each group.

**Table 6:** Histopathological scoring of the investigated groups *.

Day	Score	Experimental Groups (Number of Rats)						
		1-UINC Self-Healing	2-UIPC LHG1	3-UIPC LHG4	4-UI LHG2	5-UI4 LHG3	6-UI LHG5	7-UI LHG6
Day 3	Score 0	NF	NF	NF	NF	NF	NF	NF
	Score 1	NF	NF	NF	NF	NF	NF	NF
	Score 2	NF	NF	NF	NF	NF	NF	2
	Score 3	NF	2	2	1	2	2	1
	Score 4	3	1	1	2	1	1	NF
Day 5	Score 0	NF	NF	NF	NF	NF	NF	2
	Score 1	NF	NF	1	1	2	3	1
	Score 2	1	2	2	2	1	NF	NF
	Score 3	2	1	NF	NF	NF	NF	NF
	Score 4	NF	NF	NF	NF	NF	NF	NF
Day 7	Score 0	NF	NF	1	1	2	1	2
	Score 1	1	2	1	1	1	2	1
	Score 2	2	1	1	1	NF	NF	NF
	Score 3	NF	NF	NF	NF	NF	NF	NF
	Score 4	NF	NF	NF	NF	NF	NF	NF

* NF: Not Found, *n* = 3 in each group.

## Data Availability

The datasets generated during and/or analyzed during the current study are available from the corresponding author on reasonable request.

## Abbreviations

COL-1: collagen-1 expression, DEE: Degree of erythema and exudate, DOE: Design of Experiment, EGF: epithelial growth factor, HEC: hydroxyethyl cellulose, LHGs: licorice-based hydrogels, QbD: Quality by Design, TNF-α: Tumor Necrosis Factor alpha, UHS: ulcer healing score, UINC: Ulcer induced negative control, UIPC: Ulcer induced positive control, UPLC-ESI-MS/MS: ultra-performance liquid chromatography-electrospray tandem mass spectrometry, VEGF: Vascular endothelial growth factor, V_min_: Viscosity at minimum shear stress, WHS: wound healing score.
